# Fabrication of Hydroxyapatite with Bioglass Nanocomposite for Human Wharton’s-Jelly-Derived Mesenchymal Stem Cell Growing Substrate

**DOI:** 10.3390/ijms22179637

**Published:** 2021-09-06

**Authors:** Shamsi Ebrahimi, Yusoff Umul Hanim, Coswald Stephen Sipaut, Norsazlina binti Ahmad Jan, Sazmal E. Arshad, Siew Eng How

**Affiliations:** 1 Faculty of Engineering, Universiti Malaysia Sabah, Kota Kinabalu 88400, Sabah, Malaysia; ebrahimi_shamsi63@yahoo.com; 2 Faculty of Sciences and Natural Resources, Universiti Malaysia Sabah, Kota Kinabalu 88400, Sabah, Malaysia; hanimfaizal155@gmail.com (Y.U.H.); norsazlinaaj@gmail.com (N.b.A.J.); sazmal@ums.edu.my (S.E.A.)

**Keywords:** scaffold, nanocomposite, hWJMSCs, bioglass, osteoblast differentiation

## Abstract

Recently, composite scaffolding has found many applications in hard tissue engineering due to a number of desirable features. In this present study, hydroxyapatite/bioglass (HAp/BG) nanocomposite scaffolds were prepared in different ratios using a hydrothermal approach. The aim of this research was to evaluate the adhesion, growth, viability, and osteoblast differentiation behavior of human Wharton’s-jelly-derived mesenchymal stem cells (hWJMSCs) on HAp/BG in vitro as a scaffold for application in bone tissue engineering. Particle size and morphology were investigated by TEM and bioactivity was assessed and proven using SEM analysis with hWJMSCs in contact with the HAp/BG nanocomposite. Viability was evaluated using PrestoBlue^TM^ assay and early osteoblast differentiation and mineralization behaviors were investigated by ALP activity and EDX analysis simultaneously. TEM results showed that the prepared HAp/BG nanocomposite had dimensions of less than 40 nm. The morphology of hWJMSCs showed a fibroblast-like shape, with a clear filopodia structure. The viability of hWJMSCs was highest for the HAp/BG nanocomposite with a 70:30 ratio of HAp to BG (HAp70/BG30). The in vitro biological results confirmed that HAp/BG composite was not cytotoxic. It was also observed that the biological performance of HAp70/BG30 was higher than HAp scaffold alone. In summary, HAp/BG scaffold combined with mesenchymal stem cells showed significant potential for bone repair applications in tissue engineering.

## 1. Introduction

Scaffolding plays a significant role in tissue engineering by forming a suitable substrate for cell proliferation or differentiation, and bioceramics are known to be suitable for this purpose. Studies on bioceramics as implants began in the early 1970s. Since then, a wide variety of bioceramics have been introduced as scaffolds [1]. Bioceramics have been produced with varying shapes and phases to serve different functions in body repair. Hydroxyapatite bioceramics are among the most important studied in recent years due to their useful biocompatibility and ability to graft onto bone tissue. The many structural and chemical similarities between hydroxyapatites and the mineral phase of bone have made apatite a primary option for hard tissue repair [2,3].

One of the main concerns of nanotechnology during the last decade is the production of nano-sized particles to improve physical and chemical properties. One motivation considered by scientists to produce nano-sized materials is that most of the materials existing in natural and biological structures have nano-scale dimensions. Nanocrystalline hydroxyapatite in bone can play a significant role in its bioactivity. It is well known that the high solubility of hydroxyapatite encourages bone formation. Many studies have shown that hydroxyapatite with low crystallinity has a greater influence on the biological behavior in contact with hWJMSCs than hydroxyapatite with high crystallinity. The high concentration of calcium ions beneficially increases cellular mineralization and plays a significant role in maintaining cell growth and function. However, some studies have shown that hydroxyapatite with high crystallinity can play a significant role in modifying proliferation. In addition, [4] indicated that hydroxyapatite coating with high crystallinity and nanostructure surface on titanium implants (Ti-6AI47) plays a significant role in understanding their long-term service life as well as rapid ontogenesis on biomedical implants.

The bioactivity and biological behavior of hydroxyapatite can still be improved. As studies have shown, one way to promote bioactivity is the use of apatite to accept most of the substituted ions in its unit cell. These alternatives can be effective on surface structure and hydroxyapatite load that can affect the biological environment of the material. One interesting method for improving the bioactivity of hydroxyapatite is adding silicon to the apatite structure, since this affects the bioactivity of glass and ceramics [5]. According to previous studies, bioactive glass has been commonly used as the second phase (bioactive reinforcement) in manufacturing composites and scaffolds, commonly studied in microstructure [5]. The first bioactive glass was built in 1969 by Hench, who showed that this glass could bind with body tissues without forming blood clots around it [6]. Bioactive glass has been studied as an agent for bone regeneration [7]. Bioactive glasses are a group of materials that can be linked to living tissues of the body through the formation of a rich layer of calcium phosphate in the joints with these tissues [8]. This material can increase the expression of genes and the production of osteocalcin. Calcium silicate also has great biocompatibility and the ability to bind with living bones and soft tissue. In particular, silicon ions can form bone-like apatite [9]. Studies by [10] have also shown that nano-sized bioactive glasses have increased reactivity (and thereby enhanced stem cell proliferation and differentiation due to the presence of bioactive elements) as well as greater surface to volume ratio due to their nanostructure. It has been proven that glass nanoparticles are non-toxic and induce cell growth, which can be used in the rapid treatment of bone defects [10].

One type of glass capable of binding with bone is CaO–P_2_O_5_–SiO_2-_based glass. This glass can form crystalline hydroxycarbonate apatite (HCA) in simulated body solutions and can be used in many clinical cases which require bone building and restoration [11,12]. It is known that the incorporation of bioglass into hydroxyapatite increases the applicability of this material by allowing significant cell adhesion and osteogenic and mechanical strength characteristics [13].

In summary, nanocomposites are a new class of bonding materials at a nanometer scale that has played a decisive role in bone grafting. Since bone is an example of a nanocomposite, nanocomposite bone grafts can be expected to be more successful than other materials [14]. The purpose of this project was to improve the biological behavior of crystalline HAp by adding BG into its structure. To this end, the biocompatibility of the nanoscaffold was assessed, and the growth activity of hWJMSCs on HAp/BG scaffold with different sizes, crystallinity, and compositions was studied. The dissolution and mineralization behaviors of HAp/BG nanocrystal during the growth of hWJMSCs were also investigated.

## 2. Results and Discussion

**Figure 1:**
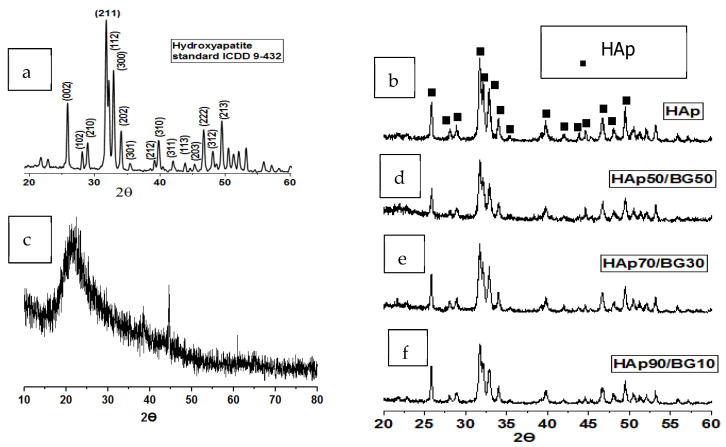
Comparison of XRD patterns. (**a**) HAp standard, (**b**) pure Hap, (**c**) BG, (**d**) HAp50/BG50, (**e**) HAp70/BG30, and (**f**) HAp90/BG10.

Figure 1 shows the X-ray diffraction patterns for the BG, HAp, and HAp/BG nanocomposite phases.

Figure 1a shows the XRD pattern for HAp standard, which includes all the peaks of hydroxyapatite. Figure 1b shows that the obtained HAp nanopowder had a pure phase and a high degree of crystallinity (80%). The prominent diffraction peaks corresponding to the 2Θ = 25.90° (002), 2Θ = 31.725° (211), 2Θ = 32.18° (112), and 2Θ = 32.86° (300) of a hexagonal structure were found to match well with ICDD 9-432 [15].

According to the XRD pattern in Figure 1c, no peak was observed in the BG samples, indicating an amorphous structure. Figure 1d–f show the XRD pattern for HAp/BG composite after sintering at 700 ℃. As seen in Figure 1d, these peaks are less intense than those of pure HAp (Figure 1b). This finding indicated that a chemical reaction had occurred between HAp and BG after the sintering process, and that Si from BG had entered into the HAp structure (Si-HAp). Also, from Table 1, both the crystallinity and crystal size of HAp in the HAp/BG composite were lower than those of pure HAp. This suggested that in the HAp/BG composite (with increased Si content), the diffraction peaks of HAp lose intensity, and HAp crystallinity and crystal size decrease. These findings are in good agreement with previous studies [16,17].

**Figure 2:**
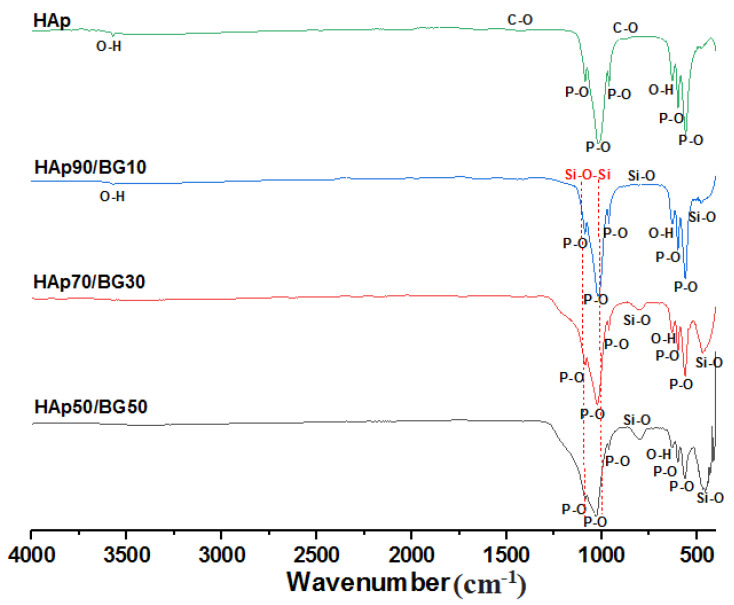
FTIR spectra of HAp and HAp/BG nano composite.

The FTIR results (Figure 2) of the HAp/BG composites were obtained based on different compositions. The vibrational models in 470, 536, 962, 1026, and 1089 cm^−1^ and absorption bands in the 603 cm^−1^ and 3000–3700 cm^−1^ ranges of all samples indicate phosphate and hydroxyl groups of hydroxyapatite structure, respectively [15]. The wavenumbers in the range between 798 cm^−1^ and 461 cm^−1^ are attributed to Si–O bonds of glass [18]. The detection of bands in the range of 1000 to 1100 shows the presence of Si–O–Si and P–O bonds, which were difficult to differentiate due to overlapping [19]. The presence of the carbonate group in the FTIR spectrum of HAp/BG composite structure was not observed. This may have been due to the decomposition of hydroxyapatite using BG in the sintering process [15]. It is interesting that, compared to HAp alone, the FTIR spectrum of HAp/BG nanocomposite showed almost no O–H bands at wave number 3570 cm^−1^. The reason for this is the decomposition of hydroxyapatite in the presence of glass, whereby silicate enters the HA structure and causes the hydroxyl group to disappear. Since glass size is in the nano range, it is likely that it encouraged the infiltration of silicate groups into the apatite network. In fact, the phase changes that occurred in a material are revealed. It is suggested that by studying the results of FTIR analysis as complementary for XRD analysis, the results will be more reliable. Similar results were obtained by previous research [16,17].

### 2.1. TEM Results for HAp/BG Nanocomposites

**Figure 3:**
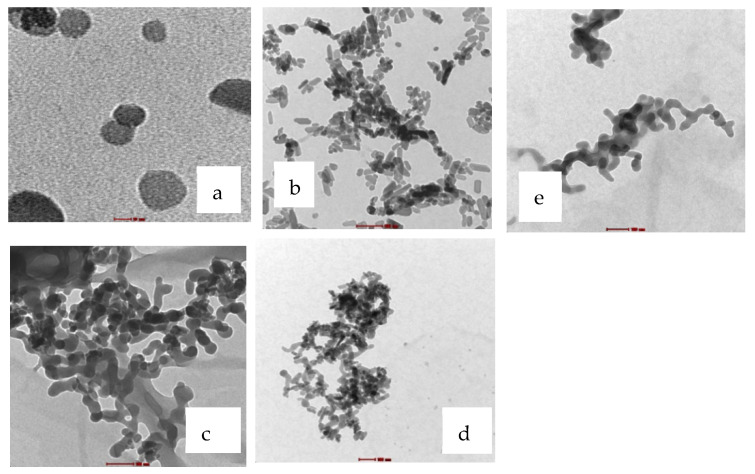
TEM results. (**a**) Hap, (**b**) HAp90/BG10, (**c**) HAp50/BG50, (**d**) HAp70/BG30, and (**e**) BG.

Figure 3 indicates transmission electron microscopy (TEM) results for HAp/BG powder without heat treatment. As can be seen, the particle size of the HAp/BG composites was less than 40 nm, which is consistent with the values of the Scherer equation (Equation (1)). From Figure 3, the size of BG particles was in the range of 50 to 150 nm, and the size of HAp was between 30 and 50 nm. At a hydrothermal treatment temperature of 180 °C, increasing the BG phase in the HAp/BG composites ratio decreased the size of HAp particles. As mentioned in the XRD results, in the HAp/BG composite, silicon was a barrier factor against HAp particle growth. In the nanocomposite with a HAp/BG ratio of 1, the HAp particles were smaller than all other ratios (Table 2). This corroborates the results of the study conducted by [16], which also found significant decreases in particle size in the Si–HAp over pure HAp [16].

It has been reported that smaller bioactive bioceramic particles are more biologically active. In other words, bone cells adjacent to very tiny bioactive particles are more proliferated. The bioactivity of bioceramics such as bioglass depends strongly on the release rate of chemical elements in the compound. The smaller the particle size, the greater the release rate of ions that influence bone-making.

### 2.2. Cell Culture Evaluation In Vitro

#### 2.2.1. Scanning Electron Microscopy Analysis of Cell Adherence and Morphology

**Figure 4:**
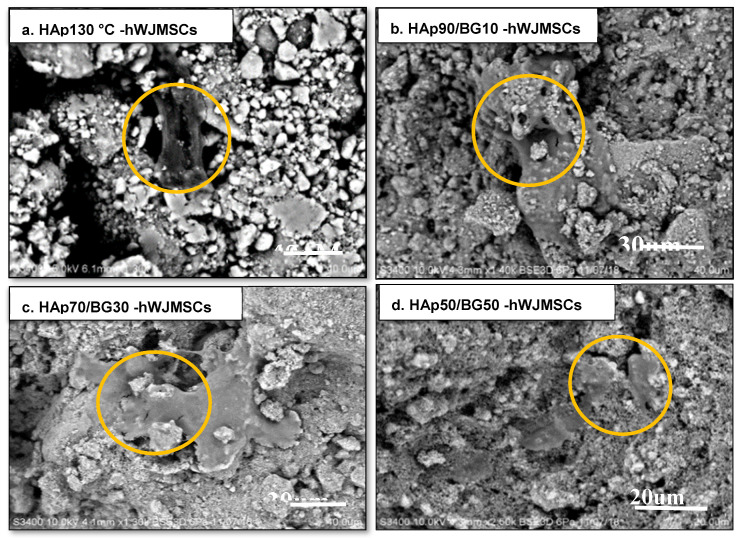
Morphology of HAp and HAp/BG-hWJMSC composites on day 6 determined by scanning electron microscope. (**a**) HAp130 °C hWJMSCs, (**b**) HAp90/BG10-hWJMSCs showing cell adherence and spreading on/within the scaffold, (**c**) HAp70/BG30-hWJMSCs with fibroblast-like shape, and (**d**) HAp50/BG50-hWJMSCs. Yellow circles show the morphology of hWJMSCs on/within each scaffold.

Samples for the morphology assessment of HAp-hWJMCs using scanning electron microscopy were taken on day 6. The morphology of HAp and HAp/BG composites showed fibroblast-like shape within the HAp surface and porosity. There was a porosity within the range of 0.777 to 18.000 μm, with the average being about 4.24 μm (Figure 4). Cell scaffold structure was observed in filopodia formation between the HAp130 °C crystalline in Figure 4a. HAp130 °C-hWJMSC morphology after 30 days (Figure 4a), and the HAp70/BG30 (Figure 4c) showed fibroblast-like shapes. As seen in Figure 4d, cell adhesion and cell spreading with 50% BG were lower than other scaffolds. Also, the structure of the scaffold with 50% BG was brittle and easily cracked. In addition, the HAp scaffold triggered h-WJMSC cell attachment by forming a bridge-like shape between the porosities. HAp70/BG30 composite was selected for prolonged culture up to 30 days. A similar result was found in previous studies [20,21].

#### 2.2.2. Cell Viability Assay Analysis

**Figure 5:**
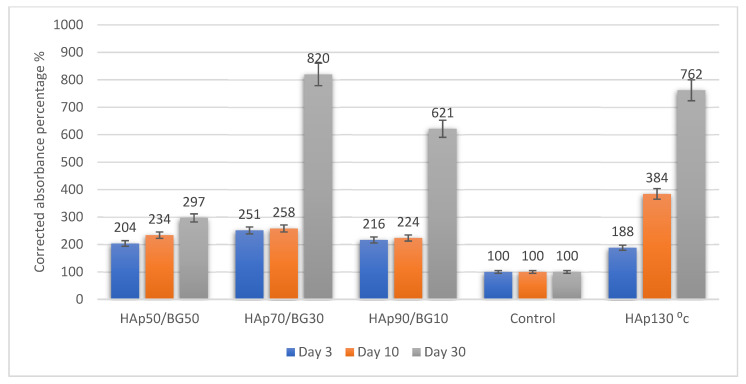
Viability of HAp/BG-hWJMSC samples on days 3, 10, and 30.

The results from the viability of cell scaffolds were expressed as the percentage of corrected absorbance against the control of HAp disc without cells. The effect of blue color turning into pinkish released from the culture of h-WJMSCs was evaluated via a Tecan^TM^ spectrophotometer, which determined the effect of each scaffold on the survival of hWJMSCs. The measurement of cell viability in this study (based on the protocol of PrestoBlue^TM^ by Thermo Fisher Scientific, Waltham, MA, USA) displayed different levels of cell viability according to the assay. The result was observed by using a Tecan^TM^ spectrophotometer (Infinite 200 Pro, Grödig, Austria) with Magellan software. The wavelength used was 570 nm, with a normalization wavelength of 600 nm. The fundamental purpose of the viability assay was to assess living cell growth in and within the scaffold materials. Figure 5 shows the viability of HAp and HAp/BG-hWJMSCs compared to the HAp scaffold without cell seeding as a control. The HAp70/BG30 scaffold showed significant increases in the viability of hWJMSCs with 8.20-fold changes compared to the control and the HAp130 °C scaffold with 7.62-fold changes at day 30. Viability analysis showed that the hWJMSCs were able to attach, spread, and proliferate safely without toxic interference from the scaffolds.

#### 2.2.3. Cell Metabolic Activity in Scaffolds

**Figure 6:**
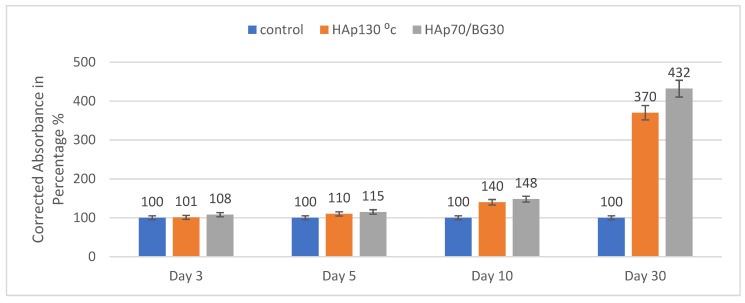
Cytotoxicity of selected cell scaffolds of HAp and HAp70/BG30 stained with MTS from day 3 until day 30.

Cell metabolic activity of hWJMSCs on HAp/BG nanocomposites was evaluated at every indicated time interval based on MTS assay, as shown in Figure 6. The HAp130 °C and HAp70/BG30 composite showed an increasing trend over 3, 5, 10, and 30 days. The highest corrected absorbance percentage was found for the HAp70/BG30 scaffold with 432% (equal to a 4.3-fold change), as shown in Figure 6. However, there were no significant differences observed between days 3 and 10 of incubation.

### 2.3. SEM-Energy Dispersed X-ray (SEM-EDX) Analysis of Osteoblast Differentiation

**Figure 7:**
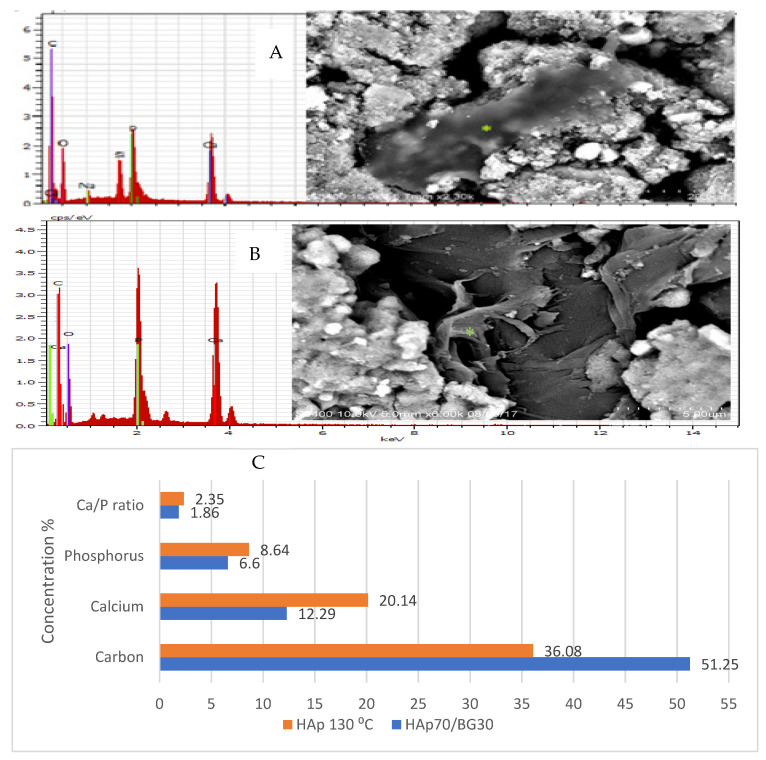
EDX analysis of hWJMSCs on HAp70/BG30 composite and HAp130 °C scaffold (**A**). EDX graph shows the calcification element composition of the cell area the in HAp70/BG30 composite scaffold. (**B**) Calcification element composition in HAp130 °C scaffold. (**C**) EDX graph of element concentration in HAp70/BG30 and HAp130 °C scaffold.

SEM-EDX analysis was conducted on the high potential composite scaffolds HAp70/BG30 and HAp130 °C to determine the concentrations of calcium (Ca), phosphorus (P), and carbon (C) in the osteo-differentiation of hWJMSCs. The results are shown in Figure 7. The concentration of C was significantly higher in HAp70/BG30 than in HAp130 °C. However, the concentrations of Ca and P were significantly higher in HAp130 °C. The Ca/P ratio of HAp70/BG30 was 1.86, close to the fabricated HAp, whereas that of HAp130 °C was 2.35.

For quantitative evaluation of cell osteogenic differentiation, hWJMSCs on the scaffolds were assessed using early-detected osteogenic differentiation assay ALP activity. Energy-dispersed x-ray (EDX) analysis is a technique used prior to discovering the major element secreted after a specific time in in vitro culture. Electron microscopy X-ray (EDX) is used for microanalysis and is a powerful technique [22].

**Figure 8:**
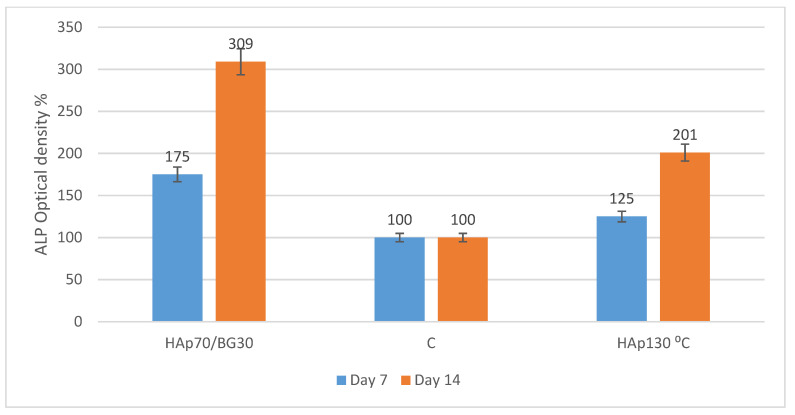
The corrected optical density of ALP activity of osteogenic differentiation hWJMSC samples on composites scaffolds at days 7 and 14.

Alkaline phosphatase (ALP) activity was measured for early osteogenic determination. Cells were harvested on days 7 and 14 for ALP analysis. As shown in Figure 8, hWJMSCs on 3D porous HAp70/BG30 demonstrated a significantly higher mean percentage corrected absorbance ALP activity (3.09-fold changes) compared to both control (without cell seeding) and also HAp130 °C (2.01-fold changes). The ALP measurements showed an increasing trend from day 7 to day 14 and suggested the potential of composite scaffolds to trigger the osteogenic differentiation of hWJMSCs.

## 3. Discussion

### 3.1. Effect of Crystal Size and Crystallinity on Cellular Response

The most important step in tissue engineering is the selection of appropriate biomaterials for scaffolding. A desirable scaffold is a model for tissue regeneration and plays a key role in the formation of the final structure of the engineered tissue and its final function. The biomaterials applied in the scaffold must support adhesion, growth, and cell proliferation both in vitro and in vivo. They should have several essential properties such as biocompatibility, biodegradability, and bioactivity [23].

Mesenchymal stem cells were cultured on all four scaffolds in a completely sterile medium. As noted, the response of cells to their surroundings in vitro depends on the type of scaffold and its preparation, as well as porosity size and surface characteristics of the scaffold. Sufficient porosity of the scaffold is essential, especially for vascularization, new tissue ingrowth, mass transport, and maintaining cell survival in a thick graft. Porosity creates favorable conditions for cellular migration vascularization among primary cells. However, because the bioglass used in this study is porous, and excessive porosity reduces mechanical strength, sintering is necessary to minimize porosity. After sintering, the composite had porosity suitable for use as a site for nucleation and the growth of apatite in the body.

In this study, HAp, BG, and HAp/BG composites had dimensions in the nanometer range. It is expected that the nanometer dimensions of bioceramic particles will have better biological performance. There are many advantages to BG and HAp nanoparticles, the most important being to increase the bioactivity of these particles. According to the theoretical principles and bioactive mechanism of these materials, it is clear that smaller particles expose greater surface area to the external environment, including cells and intercellular fluids, thereby increasing bioactivity. In other words, if two identical bioceramic compounds with the same weight are placed in hard tissue or exposed to bone cells, the bioactivity of releasing ions effective for bone making is greater in the sample with a smaller particle size. Additionally, where these materials are considered as carriers and means for transferring genes and drug into cells, the nanometer size of particles will be crucial [24]. Hydroxyapatite nanoparticles have a positive effect on the adhesion and proliferation of mesenchymal cells and osteosarcoma cells. The study by [25] showed that particles smaller than 20 nm have considerably greater bone-making ability than larger 80 nm particles [25].

This study also showed that a substrate with high crystallinity has appropriate cell adherence, viability, and differentiation. Figure 5 shows that the cell viability in the HAp50/BG50 is lower than the other scaffolds. In the HAp50/BG50 scaffold, crystallinity is low, and the structure of the scaffold was closer to the amorphous structure that leads to high solubility. Because of this higher solubility, the concentrations of ions such as calcium and phosphate in the cell culture media were higher in the HAp50/BG50 scaffold, with the result that the cellular medium was unable to refresh during the viability and cytotoxicity tests. This negative effect due to ion concentration accumulation was not observed for the stable and durable structures of HAp and HAp70/BG30. These findings are in good agreement with previous studies [26].

### 3.2. Cell Culture on HAp and HAp/BG Nanocomposite Scaffolds

HAp50/BG50-hWJMSCs (Figure 4d) showed some brittleness and cracking on the scaffold surface. In the HAp50/BG50 scaffold, glass was included in most parts of the composite, and since the strength of glass is less than hydroxyapatite, the strength of the composite was decreased. In an optimal composite of HAp and BG, HAp is packed regularly with the hydroxyapatite present in the BG surface, increasing the mechanical strength of the scaffold. The filopodia structure has driven an essential component of cell migration towards the sites [20,21] and has supported multiple problems of wound healing and of tethering cells to the extracellular matrix. The suitability of substrate conditions for cell culture is assessed with the rate of cell growth, proliferation, and migration. Electronic micrographs of the surface of HAp and HAp/BG scaffolds indicate that the nanoscaffolds had porous structures with associated pores that enhanced the attachment and growth of mesenchymal stem cells at the surface of the scaffold. As shown in Figure 4, the scaffold conditions were highly suitable for cell adhesion and migration because cells could not only adhere well to the scaffold, but were able to spread along with the pores. This confirms the biocompatibility of the scaffolds and the suitability of their surface properties for the growth and proliferation of umbilical cord mesenchymal cells. The adhesion of umbilical cord mesenchymal cells strongly depends on the size of nanoparticles. Studies have been made on the various properties of scaffolds that play important roles in the growth, morphology, and cell signaling. Cell interaction on a specific scaffold influences the vital early stages of cell activity in terms of adherence and proliferation, which regulates the cell differentiation of the cell scaffold [20]. The characteristics of the scaffold also include the size of the pores, which may assist in cell penetration, nutrients, and gas interchange [27].

In this current study, HAp70/BG30 composite sustained a significantly higher (8.2-fold) corrected absorbance reading than the other scaffolds, from day 3 until day 30. In addition, the viability on day 30 of HAp130 °C-hWJMSC was significantly (*p* ≤ 0.05) greater than the HAp, HAp90/BG10, and HAp50/BG50 scaffolds (Figure 5).

Figure 7 showed the morphology of HAp70/BG30-hWJMSC and osteo-differentiation in accordance with the EDX graph. Research by Boonrungsimana et al. (2012) demonstrated the composition of bone in terms of the Ca/P ratio. Bone is composed of inorganic elements, especially calcium (Ca) and phosphorus (P), and is enriched with carbonate content. Nonetheless, it was shown in Figure 7 that the vesicle from the apatite surface on HAp and BG subsequently mineralized the cell membrane and played an important role in extracellular matrix (ECM) activity prior to bone cell formation.

The spectrum from EDX shown in Figure 7 represented the different observations between cell attachment composition and surface without cell. Ca/P may make a higher deposit from the cell membrane area [28]. In addition, the hydroxyl and amino groups enabled an acceleration of cell differentiation into osteoblast [20].

From EDX analysis (Figure 7), this study demonstrated a specific accumulation in HAp70/BG30-hWJMSC related to altered osteo-differentiation conditions. Indeed, the result demonstrated that hWJMSCs were able to proliferate and differentiate into osteoblasts from the composition of the specific element by EDX analysis. This result is significantly different from the sample that is not a cell surface in Figure 7.

Adhesion and cell proliferation in HAp70/BG30 depend on more than hydroxyapatite alone. It is probably due to the Si–HAp phase caused by composite sintering, resulting in a good cellular response by the h-WJMSC. In the HAp/BG bioceramic, the formation of an apatite layer on the surface was faster than with HAp alone. Ref. [29] found that silicon released from the glass compound into the medium culture could increase cellular activity.

Special attention must be paid to the biological role of calcification in accordance with the detection of the elements. These traced elements have been the object of numerous studies in chemistry and medicine aimed at clarifying their role regarding the composition formation from the sample. The EDX microanalysis made it possible to project an elemental analysis on the different isotypes of calcification and expose additional information about the linkage between calcification and disease [28]. Overall, considering that hydroxyapatite and its bioglass composite are not only non-toxic but provide a suitable substrate for cell growth, differentiation, and migration, it seems HAp/BG nanocomposite scaffolds reinforced by umbilical cord mesenchymal cells can be an appropriate therapeutic strategy for the repair of extensive bone defects.

What can be deduced clearly from the adhesion and osteoblast differentiation tests is the perceived superiority of HAp/BG composite in the repair and induction of new bone formation. In studies by other researchers, there is no report comparing the bioactivity of HAp/BG composite with any of its components. Most researchers have made HAp/BG composites to enhance only mechanical properties. The results of this study showed that in addition to mechanical properties, the biological properties and bioactivity of this composite could be improved in comparison to each of its components alone [24].

## 4. Materials and Methods

### 4.1. Preparation of HAp Nanopowder

In this study, nano-size crystalline HAp powder was synthesized using the hydrothermal method. Calcium nitrate tetrahydrate (Ca(NO_3_)_2_.4H_2_O, Merck, Darmstadt, Germany) and diammonium hydrogen phosphate ((NH_4_)_2_HPO_4_, Merck, Darmstadt, Germany) with stoichiometric ratio (Ca/p = 1.67) was used for synthesis. During the precipitation, NH_3_ (Merck, Darmstadt, Germany) was applied in order to adjust the pH of the solution to a value of 10. The resulting suspension was poured into a 150 mL Teflon stainless-steel container. The hydrothermal operation was conducted at a temperature of 130 °C for 10 h. After the hydrothermal reaction, the final solution was filtered and washed with a mixture of ethanol and distilled water (volume ratio 1:1). Finally, the white hydroxyapatite powder was dried at 60 °C.

### 4.2. Preparation of BG Nanopowder

The 77S bioactive glass with a composition of 80 mol% SiO_2_ (Sigma-Aldrich, St. Louis, MO, USA): 14 mol% CaO (Sigma-Aldrich, St. Louis, MO, USA): 6 mol% P_2_O_5_ (Sigma-Aldrich, St. Louis, MO, USA) was synthesized by an acid-catalyzed sol-gel method assisted by a hydrothermal process. Hydrolysis of tetramethyl orthosilicate (TMOS, Sigma-Aldrich, St. Louis, MO, USA) and phosphoric acid (H_3_PO_4_, Sigma-Aldrich, St. Louis, MO, USA) was catalyzed with a solution of 0.1 mol L^−1^ HNO_3_ (Sigma-Aldrich, St. Louis, MO, USA). Starting with the hydrolysis of TMOS for 45 min, the other reagents were added sequentially at 60 min intervals, under constant stirring. Before reaching the gel point, the sol components were then poured into a 250 mL Teflon-lined stainless-steel autoclave and sealed. The stainless-steel autoclave was then heated in an oven at a temperature of 250 °C for 24 h. After the completion of the hydrothermal process, the autoclave was allowed to cool down to room temperature.

### 4.3. Preparation of HAp/BG Nanocomposite Powder

Nanocomposite powder was mixed with various proportions of synthesized HAp and BG powder 77s (SiO_2_, CaO, P_2_O_5_). HAp/BG nanocomposite powder was prepared in various weight ratios as listed in Table 3, under alkaline conditions (pH = 10) with stirring to make a suitable solution. Distilled water was used as the liquid phase. The final solution was transferred to a 150 mL Teflon container and was then hydrothermally treated at 180 °C for 10 h. After hydrothermal treatment, the obtained solution was filtered and then washed with distilled water. The nanocomposite powder was dried in an oven at 100 °C for 10 h, and then sintered at 700 °C for 2 h. To evaluate bioactivity, HAp/BG and HAp cement was created with dimensions of 6 mm (diameter) at 12 mm (height) and then placed into a furnace for 2 h at 700 °C.

### 4.4. Characterization

A Hitachi S3400N scanning electron microscope (SEM) equipped with an EDX (Quantex200) was used to study the morphology and chemical composition of the nanopowder samples. Transmission electron microscopy (TEM) images for evaluating particle morphology were taken using an FEI Tecnai Spirit BioTWIN at an operating voltage of 80 kV. To identify functional groups, FTIR spectra were recorded on an FTIR spectrophotometer (Perkin Elmer, Boston, MA, Spectrum 100) in the range of 400 to 4000 cm^−1^. To perform phase analysis of the nanopowders, X-ray diffraction (XRD) was carried out using an X-ray diffractometer (X’Pert Pro, PANalytical BV, Almelo, The Netherlands) with CuKa radiation source (λ = 1.54056 Å) operated at 40 kV and 30 mA. The average crystallite size was calculated from XRD data using the Debye–Scherrer approximation (Equation (1)).

(1) $$D=\frac{K\lambda }{\beta \cos\theta }$$

where β is the full width at half maximum (FWHM) of the peak at the maximum intensity and *D* is the average crystallite size.

The degree of crystallinity for HAp powder was also calculated according to Equation (2) [30].

(2) $$X_{c}\left(\%\right)=\frac{\Sigma A_{c}}{\Sigma A_{c}+\Sigma A_{A}}\times 100$$

where Σ*A_C_* + Σ*A_A_* is the sum of the area under all the HAp and HAp/BG crystalline and amorphous peaks, and Σ*A_C_* is the sum of the areas under the crystalline peaks present in the scan range between 10° and 80°.

### 4.5. In Vitro Bioactivity Preparation

#### 4.5.1. Harvest and Preparation of hWJMSCs

The primary cell line for this study was obtained from biological samples of human Wharton’s-jelly-derived mesenchymal stem cells (hWJMSCs), which were isolated from the umbilical cord collected (with ethical committee approval) from a full-term-delivery donor provided by a specific private hospital in Kota Kinabalu, Sabah, Malaysia.

The isolation procedure was performed by the Institute of Biotechnology Research (BRI) at the University Malaysia Sabah (UMS). The primary cell line was cultured in a 5% (v/v) CO_2_ incubator (NuAire, Plymouth, MN, USA) at 37 °C in standard tissue culture polystyrene (TCP) in a 25 cm^2^ flask. The cells were cultured using the basic technique of growing in Dulbecco’s Modified Eagle’s Medium (DMEM/F-12, Gibco, Waltham, MA, USA) supplemented with 10% FCS (FBS), 1% GlutaMax (100 units/mL), 1% of A-A (antibiotic–antimycotic), 1% vitamin C, and 1% GlutaMax purchased under Gibco^TM^. The primary cells were then incubated at 37 °C with 5% CO_2_ from passage zero (P0) until passage three (P3). This stabilized the heterogeneous cell culture. The primary cell line continued to be treated with HAp/BG composite scaffolds. The current media was refreshed every 2 days.

#### 4.5.2. Cell Seeding Preparation on the Scaffold

The scaffolds were UV-light sterilized using a Biosafety Cabinet (NuAire^TM^, Plymouth, MN, USA) for 1 h at both sites sequentially. The scaffolds were washed with 1x phosphate-buffered saline (1× PBS) for 2–5 min for each scaffold, before coating onto a 48-well plate and subsequent incubation in fresh media to stabilize the scaffolds before cell seeding.

Cells from a second or third passage (P2–P3) were seeded onto a 48-well plate with a density of 1 × 10^4^ cells/cm^2^. Occasionally, 7500 cells/well of 48-well plates were seeded in triplicate wells. Non-adherent cells were removed by changing the medium after 24 h of cell seeding. HAp/BG-hWJMSCs were incubated at 37 °C in 5% CO_2_ with medium changed every 2 days.

### 4.6. Cell Culture Studies In Vitro

#### 4.6.1. Cell Morphology Observation

Cell attachment and morphology were observed using a scanning electron microscope (HITACHI S-3400 N, Tokyo, Japan). The fixed samples were washed three times with 1× PBS, Gibco^TM^ post-fixed with 4% paraformaldehyde (4% PFA) in 1× PBS for 10–15 min, then washed three times with 1× PBS. Samples were then dehydrated with a graded ethanol series, soaked for 10 min each, and subsequently dried using hexamethyldisilazane solution (HMDS, Thermo Fisher Scientific, Waltham, MA, USA (Cat#TS-84770)). The assessment of the morphology of HAp/BG-hWJMCs using SEM was made on day 6^,^ and the selected potential scaffold was taken on day 30.

#### 4.6.2. Cell Viability Analysis

Evaluation of viability is essential in order to identify the cell proliferation on the biomaterial scaffolds. In this study, we assayed the HAp/BG-hWJMSC scaffolds specifically on the 3rd, 5th, 10th, and 30th days. The proliferation of hWJMSCs on the scaffolds was assessed using the cell viability assay PrestoBlue^TM^ (Thermo Fisher Scientific, Waltham, MA, USA) with a 2 h incubation time. Briefly, the cells were cultured on the scaffolds (*n* = 3) at an initial density of 1 × 10^4^ per cm^2^ (as recommended in the PrestoBlue^TM^ assay protocol, Thermo Fisher Scientific, Waltham, MA, USA). An unseeded h-WJMSC was used as a control (HA/BAG no cell).

#### 4.6.3. Cell Metabolic Activities on Scaffolds

The metabolic activity of cells in scaffolds was quantitatively evaluated by mitochondrial dehydrogenase activity using MTS assay (3-(4,5-dimethylthiazol-2-yl)-5-(3-carboxymethoxyphenyl)-2-(4-sulfophenyl)-2H-tetrazolium) from BioVision (Catalog #K300-500), according to the manufacturer’s protocol. Scaffolds were located in 48-well plates. Cells were seeded onto the scaffolds (0.75 × 10^4^ cells in 400 mL per well) as per the ratio of 10% MTS assay into cell-scaffold media and incubated for 4 h. The cell scaffolds were cultured from days 3, 5, 10, and 30 at 37 °C/5% CO_2_. After incubation for an hour, the cultures were measured at 490 nm [27].

#### 4.6.4. Elemental Composition via EDX Analysis for Osteoblast Differentiation

Assessment of the elemental composition inside the cell scaffold was conducted to compare those cell-seeded and those without cells. The evaluation of elemental composition may have significantly influenced the cell attachment and cell spreading after day 30. In addition, the assessment was performed to identify the capability of hWJMSCs to differentiate into osteoblast lineage.

#### 4.6.5. Alkaline Phosphatase (ALP) Activity Measurement for Osteoblast Differentiation

Alkaline phosphatase (ALP) activity was measured for the early osteogenic determination. Cells were harvested on day 7 and day 14 for ALP analysis. Cells were fixed in 4% paraformaldehyde (PFA) at room temperature. The manufacturer’s detailed protocol (Sigma-Aldrich, St. Louis, MO, USA, Catalog #K412-500) was followed. After the reaction was stopped by adding sodium hydroxide, the sample in the 96-well plate was measured using a microplate reader at 405 nm [31].

## 5. Conclusions

In this study, silicon-substituted hydroxyapatite with different bioglass content was synthesized by using a hydrothermal approach at 180 °C. Based on TEM analysis, the incorporation of silicon into the hydroxyapatite structure slightly decreased the size and degree of crystallinity of HAp nanoparticles. However, biological results showed that the presence of BG significantly improved the adhesion and proliferation of hWJMSCs with increased culture time to day 30. Furthermore, these scaffolds did not show any cytotoxicity effect on hWJMSCs. Importantly, the release of ions from the HAp70/BG30 scaffold was shown to be effective in bone mineralization. Taken together, these results show that HAp/BG nanocomposite will be a suitable scaffold for osteogenic differentiation in hWJMSCs.

## Figures and Tables

**Table 1:** Crystallinity and crystal size of HAp/BG composite.

Sample	Component 1 A: BG	Component 2 B: HAp	HAp/BG Crystal Size	HAp/BG Degree of Crystallinity (%)
1	0	100	38.5	80
2	10	90	37.5	77.15
3	30	70	33.12	75.84
4	50	50	31.58	67.98

**Table 2:** TEM results for HAp and its composites.

	HAp90/BG10	HAp70/BG30	HAp50/BG50	HAp
Size	31.4 nm	30.12 nm	25.1 nm	37.5 nm

**Table 3:** HAp and HAp/BG nanopowders in different ratios.

Sample	Component 1 A: BG	Component 2 B: HAp
1	0	100
2	10	90
3	30	70
4	50	50

## Data Availability

Not applicable.
